# Prostaglandin D_2_ stimulates phenotypic changes in vascular smooth muscle cells

**DOI:** 10.1038/s12276-019-0330-3

**Published:** 2019-11-18

**Authors:** Hye Sun Lee, Sung Ji Yun, Jung Min Ha, Seo Yeon Jin, Hong Koo Ha, Sang Heon Song, Chi Dae Kim, Sun Sik Bae

**Affiliations:** 1 Gene and Cell Therapy Center for Vessel-Associated Disease, Medical Research Institute, and Department of Pharmacology, Pusan National University School of Medicine, Gyungnam, 50612 Republic of Korea; 2 Department of Urology, Pusan National University Hospital, Busan, 49241 Republic of Korea; 3 Department of Internal Medicine, Pusan National University Hospital, Busan, 49241 Republic of Korea

**Keywords:** Lipid signalling, Atherosclerosis

## Abstract

Since chronic inflammation is associated with the pathogenesis of atherosclerosis, inflammatory cytokines might contribute to the phenotypic modulation of vascular smooth muscle cells (VSMCs). Tumor necrosis factor α (TNFα) facilitated the transformation of contractile VSMCs to the synthetic phenotype, as determined by the expression of marker proteins and a collagen gel contraction assay. Western blot analysis and a cyclooxygenase-2 (COX2) promoter assay revealed that TNFα stimulation resulted in the induction of COX2. The overexpression, silencing, or pharmacological inhibition of COX2 significantly affected TNFα-induced phenotypic conversion, and of the tested prostaglandins, only PGD_2_ significantly induced phenotypic conversion. ERK was significantly activated by PGD_2_ stimulation, and the pharmacological inhibition of ERK blocked the PGD_2_-induced phenotypic conversion of VSMCs. However, antagonists or agonists of PGD_2_ receptors did not affect VSMC conversion. In contrast, spontaneously dehydrated forms of PGD_2_, such as PGJ_2_, Δ^12^-PGJ_2_, and 15-d-PGJ_2_, strongly induced phenotypic conversion. A reporter gene assay showed that TNFα, PGD_2_, and 15-d-PGJ_2_ significantly activated the peroxisome proliferator-responsive element (PPRE) promoter. In addition, the overexpression or silencing of peroxisome proliferator-activated receptor δ (PPARδ) significantly influenced 15-d-PGJ_2_-induced phenotypic conversion. Finally, atherosclerotic neointima formation was significantly suppressed in mice lacking TNFα. In addition, mice fed celecoxib exhibited complete inhibition of carotid artery ligation-induced neointima formation. This study shows that PGD_2_ regulates the phenotypic conversion of VSMCs by generating an endogenous ligand of PPAR, and that this leads to neointima formation in occlusive arterial disease.

## Artherosclerosis: Switching mechanism revealed

A lipid compound that stimulates muscle cells to change type is instrumental in the development of arterial plaque formation in artherosclerosis. Sun Sik Bae at Pusan National University School of Medicine in Gyungnam, South Korea, and co-workers examined the role of inflammatory proteins in the development of artherosclerosis, a condition involving the build-up of scar tissue or ‘plaques’ on artery walls. The behavior of vascular smooth muscle cells (VSMCs) is crucial to plaque development because, triggered by inflammatory protein activity, the cells switch from contractile-type cells to faster proliferating VSMCs, accelerating plaque growth. The team found that a compound called prostaglandin D_2_, a direct by-product of inflammatory protein behavior, together with a protein regulating gene expression, are key factors triggering this VSMC change. These insights may prove valuable in developing therapies for artherosclerosis.

## Introduction

Atherosclerosis is a major leading cause of sudden death in western countries^1^, and the pathogenesis of atherosclerosis is associated with chronic inflammation resulting from interactions between lipoproteins, immune cells, and surrounding tissues, such as the endothelium and medial layers of the arterial wall. Furthermore, the migration of monocytes into vessel walls represents the initial stage of atherosclerotic plaque development^2^. Thereafter, these monocytes differentiate into macrophages, which uptake oxidized low-density lipoprotein (ox-LDL) to generate fatty streaks^3^. In addition to forming fatty streaks, vascular smooth muscle cells (VSMCs) rapidly proliferate and migrate into arterial wall lesions where they synthesize and secrete extracellular matrix proteins to form the fibrous caps of plaques^1^.

VSMCs are involved in a variety of vascular physiologies, such as vasoconstriction, vascular tone, blood pressure, and blood flow. In mature blood vessels, VSMC proliferation ceases, and the expression of specialized proteins, such as myosin heavy chain (MHC), myosin light-chain kinase (MLCK), calponin, transgelin 2 (SM22α), and ion channels, is highly elevated. Unlike cardiac or skeletal muscle cells, VSMCs retain their phenotypic plasticity. For example, contractile VSMCs undergo reversible phenotypic changes in response to environmental cues and ultimately gain the ability to proliferate and carry out their contractile function^4^. Such phenotypic conversion and the proliferation of VSMCs are observed in vascular diseases, such as atherosclerosis, arteriosclerosis, and vascular aging^5^. Recently, platelet-derived growth factor (PDGF), insulin-like growth factor-1 (IGF-1), and laminin were reported to act as environmental cues that regulate VSMC phenotype^6–8^. However, the mechanism underlying the phenotypic conversion of VSMCs in diseased states has not been elucidated.

Much evidence supports the idea that atherosclerosis is a result of chronic inflammation. Indeed, cells of the innate and adaptive immune systems, such as monocytes, macrophages, dendritic cells (DCs), B and T cells, and mast cells, are found in atherosclerotic plaque^9,10^. Chronic inflammation is closely associated with lesion progression, which is characterized by VSMC migration across the internal elastic lamina into intimal or subendothelial areas^1^. Furthermore, lesion development at this stage is influenced by inflammatory cytokines derived from interactions between monocytes/macrophages and T cells^11^.

During atherosclerosis progression, inflammatory cytokines, such as interleukin-1β (IL-1β) and tumor necrosis factor α (TNFα), are involved in vascular remodeling. For example, IL-1β deficiency suppresses the progression of atherosclerosis and outward vascular remodeling in a murine model^12–14^. In particular, vascular remodeling is modulated by TNFα, and vascular wall thicknesses in TNFα/ApoE double-knockout mice have been reported to be thinner during the later stage of atherosclerosis^15^. In addition, it has been reported that atherosclerotic lesions are smaller in TNFα/ApoE double-knockout mice^16,17^. Thus, it appears that TNFα is an important inflammatory cytokine during the progression of atherosclerosis.

It has also been reported that TNFα can induce the expression of cyclooxygenase-2 (COX2) in a variety of cell types, such as renal outer medulla cells, dorsal root ganglion cells, carcinoma cells, endometrial cells, and chondrocytes^18–22^, and that the induction of COX2 by TNFα plays a key role in VSMC proliferation^23^. COX regulates the rate-limiting step of the production of thromboxane A_2_ (TXA_2_) and prostaglandins, such as prostaglandin D_2_ (PGD_2_), prostaglandin I_2_ (PGI_2_), prostaglandin E_2_ (PGE_2_), and prostaglandin F_2α_ (PGF_2α_)^24^. Furthermore, prostaglandins have diverse effects on VSMCs; for example, they induce contraction, relaxation, and proliferation^25–28^. In particular, PGD_2_ is metabolized to generate additional inflammatory cytokines, such as prostaglandin J_2_ (PGJ_2_), Δ^12^-prostaglandin J_2_ (Δ^12^-PGJ_2_), 15-deoxy-Δ^12,14^-prostaglandin J_2_ (15-d-PGJ_2_), Δ^12^-prostaglandin D_2_ (Δ^12^-PGD_2_), and 13,14-dihydro-15-keto-PGD_2_ (DK-PGD_2_)^29,30^; of these metabolites, PGJ_2_ prostaglandins are known to be endogenous ligands of peroxisome proliferator-activated receptor (PPAR)^31,32^.

PPARs are members of a superfamily of nuclear receptors, which includes PPARα, PPARδ, and PPARγ^33^, and PPARs have been reported to play diverse roles in the vasculature. For instance, it has been reported that the smooth muscle-specific overexpression of PPARγ represses smooth muscle marker gene expression^34^, that PPARδ is upregulated during vascular lesion formation, and that its overexpression enhances VSMC proliferation^35^. Therefore, the underlying mechanisms of PPARs in the vasculature remain unclear. In the present study, we investigated critical inflammatory cytokines that determine the phenotypic switching of VSMCs and attempted to define the molecular mechanisms underlying inflammatory cytokine-mediated signaling pathways.

## Materials and methods

### Animals

Mice lacking *TNFα* (*TNFα*^*−/−*^, B6.129S-*Tnf*^*tm1Gkl*^/J mice) and *ApoE* (*ApoE*^−*/*−^, C57B6.129P2-*Apoe*^*tm1Unc*^/J mice) were purchased from The Jackson Laboratory (Bar Harbor, Maine, USA). *TNFα*^*−/−*^ mice were crossed with *ApoE*-deficient mice (*ApoE*^*−/−*^) to generate mice heterozygous at both loci. *TNFα*^*+/−*^*ApoE*^*−/−*^ mice were intercrossed to produce *TNFα*^*+/+*^*ApoE*^*−/−*^ and *TNFα*^*−/−*^*ApoE*^*−/−*^ littermates. Animals were housed under specific pathogen-free conditions. All animal procedures were performed in accordance with our institutional guidelines for animal research and were approved by our institutional animal care and use committee (PNU-2016-1195). The investigation conformed with the Guide for the Care and Use of Laboratory Animals published by the US National Institutes of Health (NIH Publication No. 85-23, revised 1996).

### Cell preparation and cell culture

VSMCs were isolated from 4-week-old male Sprague-Dawley rats by using a tissue explantation method. Briefly, rats were euthanized by intravenous ketamine (100 mg/kg) injection and perfused with phosphate-buffered saline (PBS) for 5 min. The thoracic aorta was aseptically isolated, and the surrounding fat and connective tissues were discarded. Vessels were cut longitudinally, and the lumen sides were scraped with a razor blade to remove the intima. Vessels were cut into 3–5-mm lengths and explanted the lumen side down onto collagen-coated culture dishes. Seven days after explantation, the tissue fragments were discarded, and sprouted VSMCs (referred to as P0-stage VSMCs) were collected.

### Preparation of contractile phenotype VSMCs and phenotypic conversion

To prepare contractile VSMCs, P0-stage VSMCs were differentiated as described previously^8^. The differentiation of VSMCs was verified by western blotting for SMC marker proteins, namely, MHC, MLCK, SM22α, calponin, and smooth muscle actin (SMA). Contractile VSMCs were cultured with medium containing the indicated inflammatory cytokines for 4 days. To examine the facilitation of phenotypic conversion by PPARδ overexpression, cells were incubated with the indicated cytokines for 2 days.

### Immunocytochemistry

For immunocytochemistry, cells were washed with ice-cold PBS and fixed with 4% paraformaldehyde for 10 min. The cells were permeabilized with 0.2% Triton X-100, incubated with the indicated primary antibodies for 1 h, and then treated with Cy3- or Alexa Fluor 488-conjugated secondary antibodies for 30 min. The samples were mounted with anti-fading reagent (2% n-propyl gallate in 80% glycerol/PBS solution), and images were obtained by using a confocal microscope (FV1000-ZDC, Olympus, Tokyo Japan).

### Western blotting

Cell lysates were subjected to sodium dodecyl sulfate polyacrylamide gel electrophoresis on 10% polyacrylamide gels under reducing conditions. The proteins were transferred to nitrocellulose membranes, which were immunoblotted by using the indicated primary antibodies and IRDye-conjugated secondary antibodies (Li-COR Biosciences, Lincoln, NE, USA). The western blots were developed by using the Odyssey system (Li-COR Biosciences).

### Collagen gel contraction assay

VSMCs were isolated by trypsin digestion and resuspended in serum-free DMEM (1 × 10^6^ cells/ml). The cell suspensions were mixed on ice with collagen gel solution (8 mg/ml collagen type I in 2× PBS, pH 8.0) to give 5 × 10^5^ cells/ml and 4 mg/ml collagen gel solution. One hundred microliters of VSMC–collagen gel mixture was added to 12-well plates, which were then incubated for 30 min at 37 °C to enable polymerization. The gels were then floated in serum-free DMEM for 5 h, and angiotensin II (AngII, 1 μM) was added to initiate contraction, while images were captured by using a digital charge-coupled device camera. Collagen gel contraction was defined as a decrease in the gel area as determined by Scion Image software (compliments of Scion Corporation, Frederick, MD; http://www.scioncorp.com). Relative gel areas were obtained by dividing the areas by the initial areas.

### Plasmids and promoter assay

pGL3-SMA and pGL3-SM22α were kindly provided by Dr. Gary K. Owens (University of Virginia). pGL3-myocardin was prepared as described previously^8^. pGL3-PPRE and pCDNA3.1-PPARδ were provided by Dr. Jang Hyun Choi (UNIST, Ulsan, Korea). Human COX2 cDNA was subcloned into the *BamHI*/*EcoRI* site of the pMIGR2 vector. To assess promoter activity, we used a dual-luciferase reporter assay system. VSMCs were plated in 12-well plates and cotransfected with the luciferase reporter constructs and the renilla luciferase plasmids by using Lipofectamine 2000 (Invitrogen, Carlsbad, CA, USA). Each well contained 0.88 μg of luciferase reporter plasmid, 0.8 μg of expression vector, and 80 ng of renilla luciferase plasmid. The medium was replaced with fresh medium 7 h post transfection, and the cells were lysed and assayed for luciferase activity 24 h post transfection. The protein extracts (20 μl) were analyzed by using a Glomax^TM^ 20/20 luminometer (Promega, WI, USA).

### Short-hairpin RNA and constructs

To silence COX2 and PPARδ, shCOX2 (5′-CCG GTA AGA CAG ATC AGA AGC GAG GAC TCG AGT CCT CGC TTC TGA TCT GTC TTT TTT TG-3′) and shPPARδ (5′-CCG GTG CAA GCC CTT CAG TGA CAT CAC TCG AGT GAT GTC ACT GAA GGG CTT GCT TTT TG-3′) oligonucleotides with an *AgeI* site at the 5′-end site and an *EcoRI* site at the 3′ end were designed, and then sense and antisense oligonucleotides were synthesized (XENOTECH, Daejeon, Korea). Both complementary oligonucleotides were mixed, heated at 98 °C for 5 min, and cooled to room temperature. The annealed nucleotides were subcloned into the *AgeI*/*EcoRI* sites of the pLKO.1 lentiviral vector.

### Lentiviral knockdown

For gene silencing, HEK293-FT packaging cells (Invitrogen) were grown to ~70% confluence in six-well plates. The cells were triple transfected with 6 μg of the pLKO.1 lentiviral construct, 1 μg of Δ8.9, and 1 μg of pVSV-G by using a calcium phosphate method. The medium was replaced with fresh medium 8 h post transfection. Lentiviral supernatants were harvested 24 h post transfection and passed through 0.45-μm filters. Cell-free viral culture supernatants were used to infect contractile VSMCs in the presence of 8 μg/ml polybrene (Sigma-Aldrich, St. Louis, MO, USA). Additional rounds of infection were performed 48 and 72 h post transfection. Infected cells were isolated by selection by using 10 μg/ml puromycin for 2 days.

### Carotid artery ligation, induction of atherosclerosis, and immunohistochemistry

To induce neointima formation, the left common carotid artery of male mice (6 weeks old) was ligated proximal to the bifurcation, and 4 weeks later, both the left and right common carotid arteries were isolated. To treat the mice with celecoxib, the mice were fed a D12450B diet containing 1500 ppm of celecoxib (Central Lab. Animal Inc., Seoul) for 2 weeks before ligation and fed a diet containing celecoxib for an additional 4 weeks. Atherosclerosis was induced by feeding male mice (8 weeks old) a high-fat western-type diet containing 1.25% cholesterol (Research Diets, D12108) for 15 weeks. After killing, the mice were perfused with PBS, and the isolated carotid arteries and aortas were fixed in 4% paraformaldehyde at 4 °C overnight and embedded in paraffin for immunohistochemistry. Five-micrometer sections of each block were either stained with H&E or the indicated primary antibodies. Staining was visualized by using a MIRAX MIDI Versatile Digital Slide Scanner (Carl Zeiss, Jena, Germany) or by confocal microscopy (Olympus). For oil red O staining, aortic tissues were stained with oil red O solution (0.5% in isopropyl alcohol) for 2 h and washed with distilled water four times. For quantification, oil red O was extracted with isopropyl alcohol (1 ml), and the absorbance was measured at 518 nm.

### Statistical analysis

The data were plotted and analyzed by using GraphPad Prism. Unpaired Student’s *t* test (two tails) was used to determine the significance of intergroup differences. Multiple sets of data were analyzed by analysis of variance (one-way ANOVA) and Tukey’s multiple comparison test. The results are expressed as the means ± SEMs, and *P* values less than 0.05 were considered significant.

## Results

### Phenotypic changes in VSMCs induced by TNFα

**Fig. 1:**
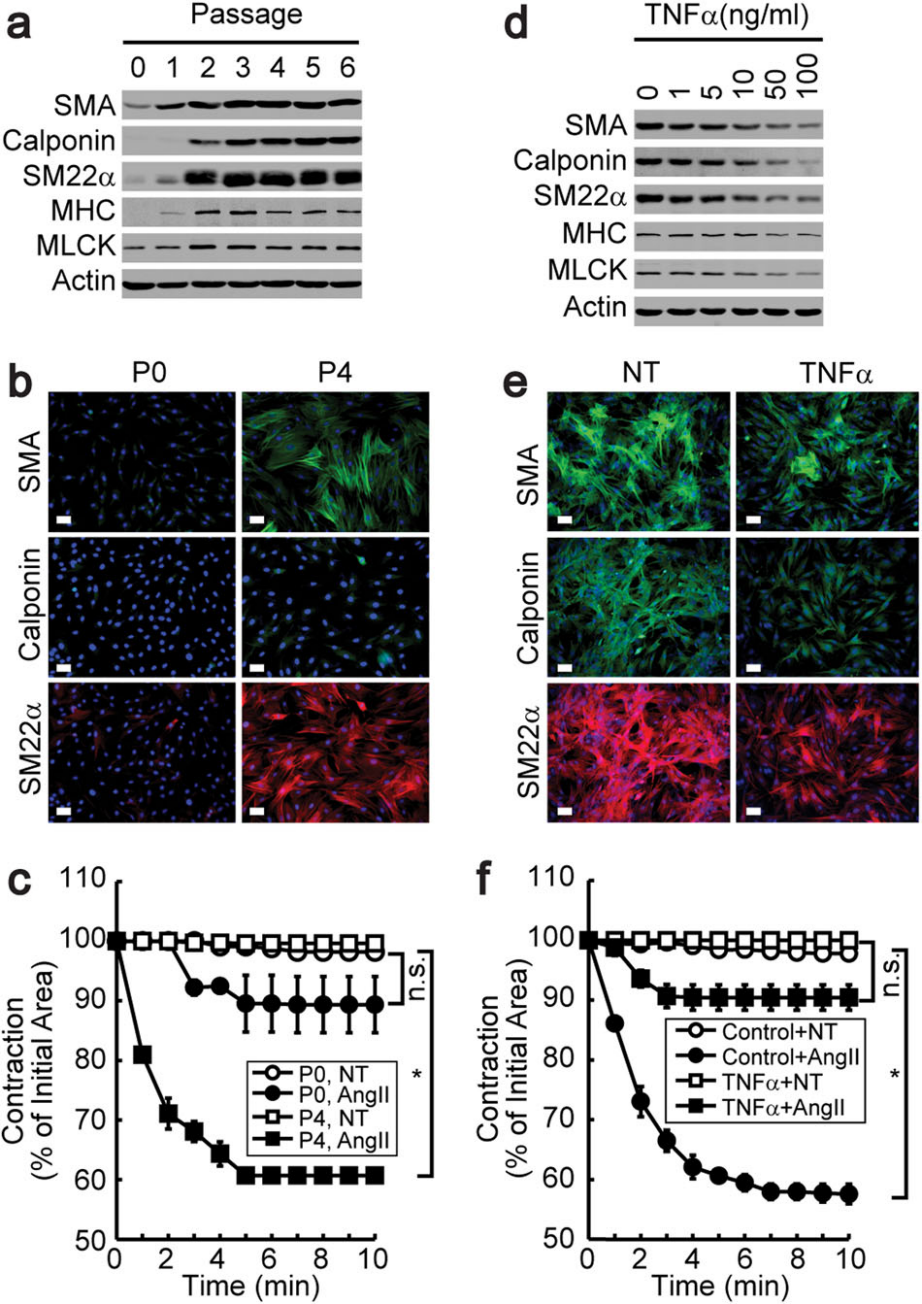
Phenotypic conversion of contractile VSMCs by TNFα. Synthetic VSMCs isolated from rat aortic tissue fragments were explanted onto laminin-coated plates. The expression of contractile marker genes was assessed by western blot analysis (**a**) or immunocytochemistry (**b**) at the indicated passages. Magnification, 40×. Bar, 100 μm. **c** Cells from each passage were embedded in collagen gel beads and stimulated with AngII. Time-lapse images were recorded digitally, and contractions are expressed as the percentage of the initial area (*n* = 3). *P < 0.05 compared with the no- treatment (NT) group. n.s., not significant. P4-stage VSMCs were stimulated with TNFα for 4 days at the indicated doses, and the expression of contractile marker genes was assessed by western blot analysis (**d**) and immunocytochemistry (**e**). Magnification, 40×. **f** Contractile VSMCs were stimulated with TNFα for 4 days, and AngII-induced collagen gel contraction was analyzed as described above (*n* = 3). *P < 0.05 compared with the no-treatment (NT) group. n.s. not significant. The results are presented as the means ± SEM. One-way ANOVA and Tukey’s multiple comparison test were used to determine the *P* values. The asterisks indicate statistical significance (*P* < 0.05).

Since VSMC sprouting after the explantation of aortic tissue fragments on collagen-coated plates is indicative of the synthetic phenotype, we employed a previously described differentiation protocol^8^. Plating VSMCs on laminin-coated plates initiated differentiation, as shown in Fig. 1a, b. P0-stage VSMCs did not respond to AngII stimulation, whereas P4-stage VSMCs rapidly contracted in response to AngII stimulation (Fig. 1c). However, the differentiation of established cell lines, such as A10 cells, did not result in contraction in response to AngII stimulation (Supplementary Fig. S1). The treatment of contractile VSMCs (P4 stage) with TNFα significantly reduced the expression of SMC marker proteins (Fig. 1d, e) and the promoter activity of myocardin, SMA, and SM22α (Supplementary Fig. S2). Furthermore, TNFα treatment resulted in the loss of AngII-dependent contraction (Fig. 1f).

### COX2 was required for TNFα-induced phenotypic changes in VSMCs

**Fig. 2:**
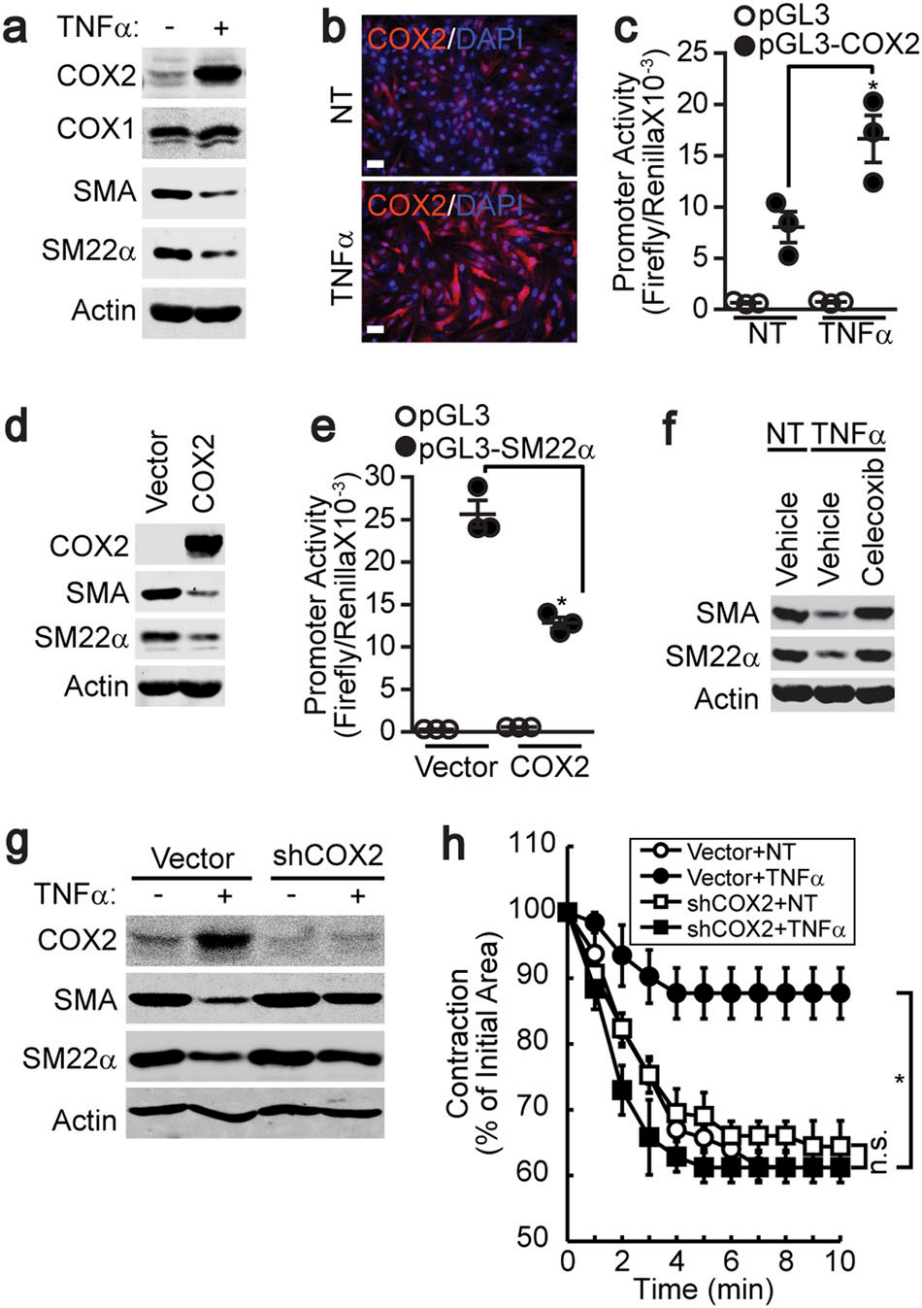
COX2 was required for TNFα-induced VSMC phenotypic changes. P4-stage VSMCs were stimulated with TNFα for 4 days, and the expression levels of COX2 and contractile marker genes were assessed by western blot analysis (**a**) and immunocytochemistry (**b**). Magnification, 40×. Bar, 100 μm. **c** The COX2 promoter was subcloned into the pGL3 vector and was transfected into P4-stage VSMCs (*n* = 3). The TNFα-induced activation of the COX2 promoter was assessed as described in the “Materials and methods” section. COX2 was ectopically expressed in P4-stage VSMCs, and the expression levels of COX2, SMA, and SM22α (**d**) and SM22α promoter activity (**e**) were examined (*n* = 3). **f** P4-stage VSMCs were pretreated with a selective COX2 inhibitor (celecoxib), and TNFα-dependent gene expression changes were examined. COX2 was silenced in P4-stage VSMCs, and TNFα-dependent gene expression (**g**) and the loss of contractility were evaluated (*n* = 3) (**h**). *P < 0.05 compared with the untreated group. n.s. not significant. The results are presented as the means ± SEM. One-way ANOVA and Tukey’s multiple comparison test were used to determine the *P* values. The asterisks indicate statistical significance (*P* < 0.05).

COX2 expression was significantly reduced during the differentiation of VSMCs from the synthetic to the contractile phenotype (Supplementary Fig. S3a), whereas the stimulation of contractile VSMCs with TNFα resulted in the induction of COX2 (Fig. 2a, b). In addition, the stimulation of VSMCs with TNFα strongly enhanced the promoter activity of COX2 (Fig. 2c). Furthermore, ectopic COX2 expression significantly reduced the expression of marker proteins, such as SMA and SM22α, and the promoter activity of SM22α (Fig. 2d, e). In addition, the pharmacological inhibition of COX2 by celecoxib (10 μM) markedly inhibited the TNFα-induced phenotypic conversion of contractile VSMCs (Fig. 2f). The silencing of COX2 blunted the TNFα-induced phenotypic conversion of contractile VSMCs and preserved the expression of contractile phenotype marker proteins and AngII-dependent contraction (Fig. 2g, h, Supplementary Fig. S3b).

### PGD_2_ regulated the phenotypic changes in VSMCs independently of the DP_1_ and DP_2_ receptor signaling pathways

**Fig. 3:**
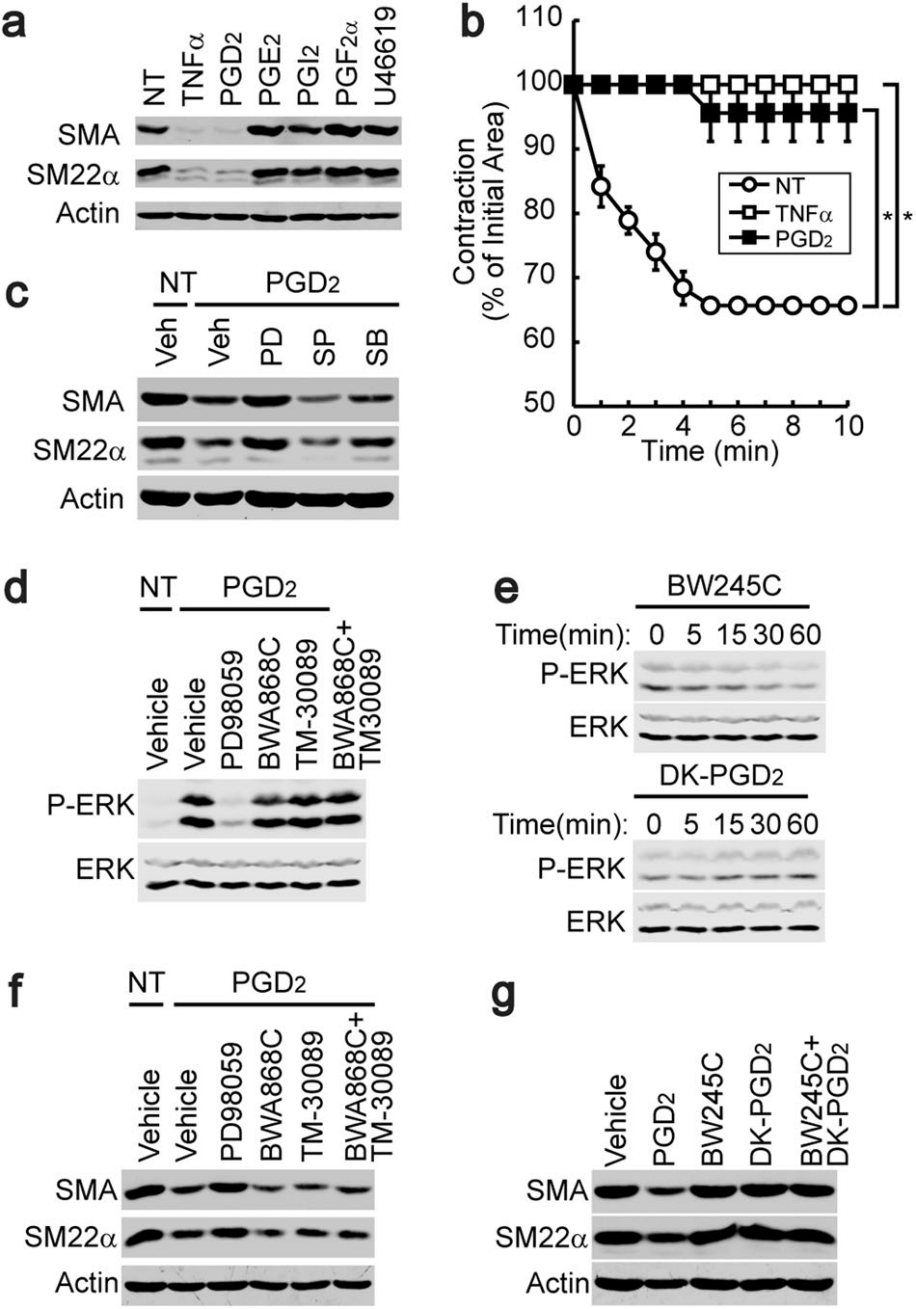
DP_1_- and DP_2_-independent VSMC phenotypic changes induced by PGD_2_. **a** P4-stage VSMCs were stimulated with the indicated prostanoids or a thromboxane analog (U46619) for 4 days. Cell lysates were examined for the expression of SMA and SM22α. **b** P4-stage VSMCs were stimulated with TNFα or PGD_2_ for 4 days, and AngII-dependent contraction was analyzed (*n* = 2). *P < 0.05 compared with the no-treatment (NT) group. **c** P4-stage VSMCs were pretreated with the indicated inhibitors (PD, PD98059; SP, SP600125; SB, SB203580), and PGD_2_-dependent changes in the expression of SMA and SM22α were assessed. **d** P4-stage VSMCs were pretreated with a MEK, DP_1_, or DP_2_ inhibitor (PD98059, BWA868C, or TM-30089) and then stimulated with PGD_2_. Cell lysates were analyzed for ERK activation. **e** P4-stage VSMCs were stimulated with a DP_1_ or DP_2_ agonist (BW245C (upper panel) or DK-PGD_2_ (lower panel)), and ERK activation was assessed. **f** P4-stage VSMCs were pretreated with a DP_1_ or DP_2_ antagonist (BWA868C or TM-30089), and PGD_2_-dependent gene expression changes in SMA and SM22α were examined. **g** P4-stage VSMCs were stimulated with a DP_1_ or DP_2_ agonist (BW245C (upper panel) or DK-PGD_2_ (lower panel)), and the expression levels of SMA and SM22α were assessed. The results are presented as the means ± SEM. One-way ANOVA and Tukey’s multiple comparison test were used to determine the *P* values. The asterisks indicate statistical significance (*P* < 0.05).

Since COX2 plays a key role in the rate-limiting step of prostaglandin synthesis, we examined the effect of prostaglandins on the expression of SMC marker proteins. The stimulation of contractile VSMCs with PGD_2_ (10 μM) significantly downregulated the expression of SMC marker proteins, whereas other prostaglandins had no effect (Fig. 3a, Supplementary Fig. S4a, b). In addition, the stimulation of contractile VSMCs with PGD_2_ abrogated AngII-dependent contraction (Fig. 3b). The stimulation of contractile VSMCs with PGD_2_ significantly activated the ERK signaling pathway in a time-dependent manner (Supplementary Fig. S4c), and the inhibition of ERK blunted PGD_2_-induced VSMC phenotypic changes (Fig. 3c). DP_1_ and DP_2_, which couple to G_s_ and G_i_, respectively, regulate cAMP production and are known to be PGD_2_ receptors^36^. However, the treatment of contractile VSMCs with PGD_2_, a DP_1_ agonist (BW245C, 10 μM), or a DP_2_ agonist (DK-PGD_2_, 10 μM) did not change cAMP production (Supplementary Fig. S5a–d). In addition, the expression of DP_1_ and DP_2_ was not observed in either synthetic or contractile VSMCs (Supplementary Fig. S5e). PGD_2_ did not induce calcium mobilization, which is mediated by the activation of G_q_ and phospholipase C-β (Supplementary Fig. S5f–h). Since the ERK signaling pathway is important for the PGD_2_-dependent suppression of SMC marker gene expression, we examined whether the PGD_2_ receptors DP_1_ and DP_2_ are involved in the activation of the ERK signaling pathway. As shown in Fig. 3d, the inhibition of MEK by PD98059 (10 μM) completely blocked the PGD_2_-induced activation of ERK. However, the inhibition of DP_1_ (BWA868C, 10 μM) or DP_2_ (TM-30089, 10 μM) or the simultaneous inhibition of DP_1_ and DP_2_ did not block the PGD_2_-induced activation of ERK. In addition, a selective agonist of DP_1_ (BW245C, 10 μM) or DP_2_ (DK-PGD_2_, 10 μM) did not induce the activation of ERK (Fig. 3e). Furthermore, neither agonists nor antagonists of DP_1_ and DP_2_ affected the expression of SMC marker proteins (Fig. 3f, g).

### The PGJ_2_ series of PGD_2_ metabolites affected VSMC phenotypes

**Fig. 4:**
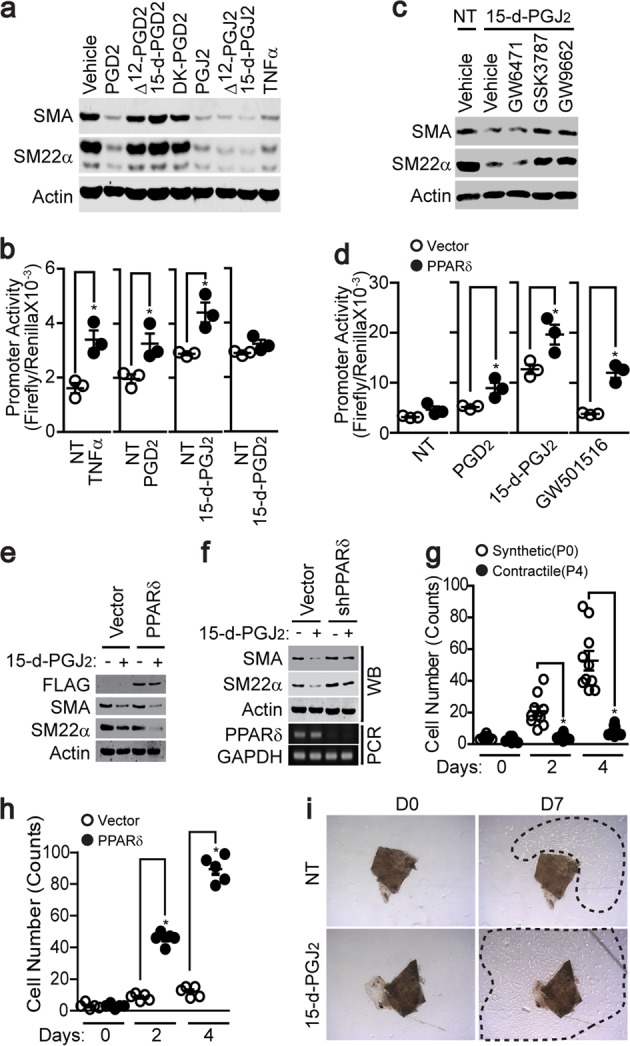
PPARδ was required for PGD_2_- and 15-d-PGJ_2_-dependent VSMC phenotypic changes. **a** P4-stage VSMCs were stimulated with the indicated PGD_2_ metabolites, and the expression levels of SMA and SM22α were assessed by western blot analysis. **b** The promoter activity of PPRE was verified in the presence of the indicated stimuli (*n* = 3). **c** P4-stage VSMCs were pretreated with a PPARα, PPARδ, or PPARγ antagonist (GW6471, GSK3787, or GW9662) and then stimulated with 15-d-PGJ_2_. The expression levels of SMA and SM22α were assessed by western blot analysis. **d** Contractile VSMCs were infected with a retrovirus carrying either vector or the PPARδ construct, and the promoter activity of PPRE was verified in the presence of the indicated stimuli (*n* = 3). GW501516 (10 μM) was used as a positive control for the PPARδ agonist. **e** FLAG-tagged PPARδ was expressed in P4-stage VSMCs, and the expression levels of SMA and SM22α were assessed after stimulation with 15-d-PGJ_2_. **f** PPARδ was silenced in P4-stage VSMCs, and the expression of SMA and SM22α was assessed after cells were stimulated with 15-d-PGJ_2_. **g** The proliferation of P0- or P4-stage VSMCs was measured (*n* = 10). **h** Vector or PPARδ was expressed in P4-stage VSMCs, and proliferation was measured (*n* = 5). **i** Aortic tissues were isolated, the endothelial and adventitial layers were discarded, and the aortic tissue fragments were placed on collagen-coated plates and then stimulated with 15-d-PGJ_2_ for 7 days. Proliferating VSMCs are indicated by dashed lines. The results are presented as the means ± SEM. One-way ANOVA and Tukey’s multiple comparison test were used to determine the *P* values. The asterisks indicate statistical significance (*P* < 0.05).

PGD_2_ generates inflammatory metabolites, such as Δ^12^-PGD_2_ and 15-d-PGJ_2_^29,30^. Since our results showed that DP_1_ and/or DP_2_ receptors did not affect VSMC phenotypic changes, we hypothesized that PGD_2_ metabolites are involved in the phenotypic modulation of VSMCs. As shown in Fig. 4a, the PGJ_2_ series of PGD_2_ metabolites significantly induced VSMC phenotypic changes, whereas metabolites of other series had no effect. In addition, PGJ_2_ series metabolites significantly induced ERK activation, whereas other series of metabolites did not (Supplementary Fig. S6). Since PGD_2_ and PGJ_2_ are endogenous ligands of PPAR^31,32^, we explored the effect of PGD_2_ metabolites on PPRE promoter activity. As shown in Fig. 4b, TNFα, PGD_2_, and 15-d-PGJ_2_ significantly elevated PPRE promoter activity, whereas 15-d-PGD_2_, which did not induce VSMC phenotypic changes, had no effect. In addition, VSMCs mainly expressed PPARδ and PPARγ (Supplementary Fig. S7a, b); the selective inhibition of PPARδ (GSK3787, 10 μM) and PPARγ (GW9662, 10 μM) suppressed 15-d-PGJ_2_-induced phenotypic changes in contractile VSMCs, whereas the inhibition of PPARα (GW6471, 10 μM) had no effect (Fig. 4c). Since 15-d-PGJ_2_ significantly activated the PPARδ-dependent activation of the PPRE promoter (Fig. 4d), we investigated the effect of 15-d-PGJ_2_ in the presence or absence of PPARδ. The overexpression of PPARδ significantly enhanced contractile VSMC phenotypic changes in the presence of 15-d-PGJ_2_ (Fig. 4e). Furthermore, the silencing of PPARδ inhibited 15-d-PGJ_2_-induced contractile VSMC phenotypic changes (Fig. 4f). The proliferation of contractile VSMCs was significantly lower than that of synthetic VSMCs (Fig. 4g), whereas the proliferation of contractile VSMCs was enhanced by the overexpression of PPARδ (Fig. 4h). In addition, the stimulation of abdominal aortic fragments with 15-d-PGJ_2_ facilitated VSMC sprouting ex vivo (Fig. 4i).

### Atherosclerotic neointima formation was attenuated in mice lacking TNFα

**Fig. 5:**
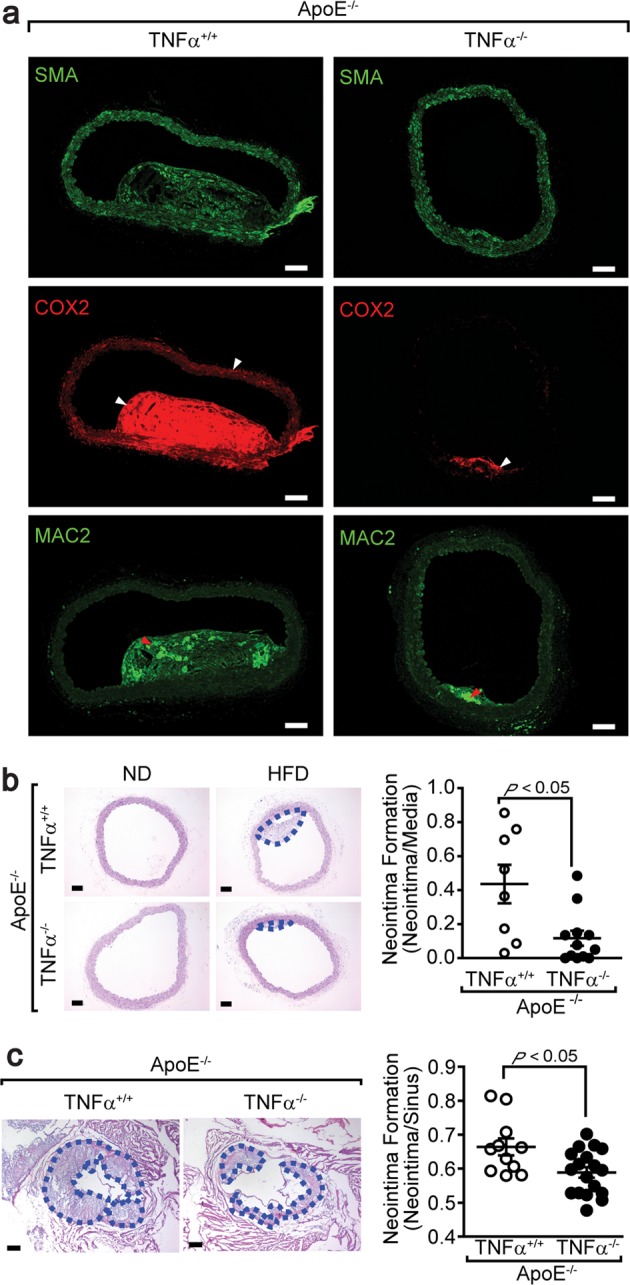
Suppression of atherosclerosis in mice lacking *TNFα*. **a** Mice lacking both *ApoE* and *TNFα* were fed a high-fat diet for 15 weeks. Aortic tissues were isolated and stained with the indicated antibodies. The white and red arrows indicate COX2 expression and macrophages, respectively. Bar, 25 μm. The mice were fed either a normal or high-fat diet for 15 weeks. Aortas (**b**) (*ApoE*^*−/−*^*TNFα*^*+/+*^ mice, *n* = 8; *ApoE*^−*/−*^*TNFα*^*−/−*^ mice, *n* = 12) and aortic sinuses (**c**) (*ApoE*^−*/−*^*TNFα*^*+/+*^ mice, *n* = 11; *ApoE*^–*/*–^*TNFα*^*–/*–^ mice, *n* = 18) were stained with hematoxylin and eosin, and the neointimal areas were measured. The dashed blue lines indicate neointimal tissues. Bar, 50 μm. The results are presented as the means ± SEM. Unpaired Student’s *t* test (two tails) was used to determine the *P* values.

To assess the role of TNFα in the regulation of the VSMC phenotype in vivo, mice lacking both *ApoE* and *TNFα* (*ApoE*^*−/−*^*TNFα*^*−/*−^) were fed a high-fat western diet, and neointima formation was examined. As shown in Fig. 5a, mice fed a high-fat diet exhibited significantly induced COX2 expression in the inflamed medial layers and plaque lesions, whereas COX2 expression was abolished in mice lacking *TNFα*. Macrophages were observed only in plaque lesions in both genotypes. Neointima formation was significantly lower in *ApoE*^*−/−*^*TNFα*^*−/−*^ mice than in *ApoE*^*−/−*^*TNFα*^*+/+*^ mice (Fig. 5b). To avoid inadvertent errors caused by the locations of the sections, we also examined neointima formation in the aortic sinuses. As shown in Fig. 5c, neointima formation in mice lacking *TNFα* was significantly reduced.

### Carotid artery ligation-induced neointima formation was attenuated in mice lacking TNFα or fed celecoxib

**Fig. 6:**
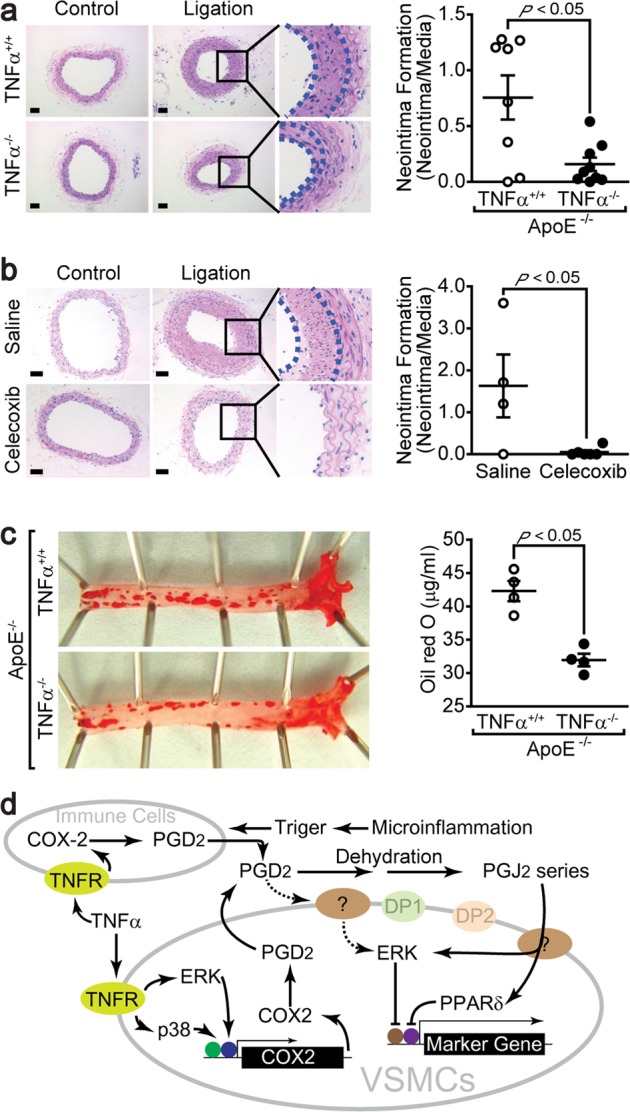
Suppression of neointima formation by COX2 inhibition. **a** The carotid arteries of mice lacking TNFα were ligated for 4 weeks (*ApoE*^−*/*−^*TNFα*^*+/+*^ mice, *n* = 8; *ApoE*^*−/−*^*TNFα*^−*/−*^ mice, *n* = 9). Representative photomicrographs of cross sections of carotid arteries stained with hematoxylin and eosin are shown. The dashed line defines the medial and neointimal layers. Bar, 25 μm. **b** The mice were fed a diet containing a selective COX2 inhibitor (celecoxib) for 2 weeks before carotid artery ligation and fed a diet containing celecoxib for an additional 4 weeks (saline, *n* = 4; celecoxib, *n* = 6). Aortic tissues were isolated, and cross sections from 2 mm distal to the ligation were stained with hematoxylin and eosin. The dashed lines define the medial and neointimal layers. Bar, 25 μm. **c** The mice were fed a high-fat diet for 15 weeks, abdominal aortas were isolated and stained with oil red O, and lipid accumulation was quantified by extracting oil red O with isopropyl alcohol (*n* = 4). **d** A schematic representation of the PGD_2_-mediated phenotypic modulation of VSMCs by PPARδ. The results are presented as the means ± SEM. Unpaired Student’s *t* test (two tails) was used to determine the *P* values.

We next adapted the carotid artery ligation model to determine whether TNFα and COX2 are required for neointima formation. In control mice, the ligation of the left common carotid artery distal to the aortic arch near the bifurcation markedly induced neointima formation, whereas neointima formation was significantly lower in mice lacking TNFα (Fig. 6a). In addition, mice fed the selective COX2 inhibitor celecoxib exhibited completely blocked carotid artery ligation-induced neointima formation (Fig. 6b). Overall, plaque formation was significantly decreased in mice lacking *TNFα*, as assessed by oil red O staining (Fig. 6c).

## Discussion

The present study demonstrates that TNFα regulates the dedifferentiation of contractile VSMCs to the synthetic phenotype via the induction of COX2 and the production of prostaglandins, such as PGD_2_ and PGJ_2_. In addition, it shows that the loss of TNFα and the inhibition of COX2 suppresses the induction of intimal thickening in mice. Consequently, we propose that the PGD_2_-mediated activation of PPARδ is a potential target for the inhibition of neointima formation.

It has been reported that vascular remodeling is closely associated with chronic inflammation^9,37^. Therefore, it is possible that inflammatory cytokines play pivotal roles in vascular remodeling, particularly in the phenotypic switching of VSMCs. In particular, TNFα has been implicated in the pathogenesis of atherosclerosis; for example, the disruption of TNFα has been reported to delay the progression of atherosclerosis and to reduce lesion sizes^15,17^. The production of TNFα by cells of the hematopoietic lineage appears to be important because the transplantation of bone marrow from mice lacking TNFα has been found to reduce the severity of atherosclerosis^16^. Likewise, our results show that atherosclerotic lesion size was significantly reduced in mice lacking TNFα (Fig. 5). One of the major roles of TNFα seems to be involved in VSMC phenotypic changes. For instance, the stimulation of contractile VSMCs suppresses the expression of smooth muscle marker genes and results in the loss of contractile function in smooth muscle cells (Fig. 1). The consequence of TNFα-induced VSMC phenotypic changes is VSMC hyperproliferation since synthetic VSMCs grow faster than contractile VSMCs in vitro (Fig. 4g)^8^. Hence, we suggest that TNFα stimulates phenotypic conversion in smooth muscle, thereby enhancing VSMC proliferation and causing neointima formation.

Although the mechanisms underlying the TNFα-mediated phenotypic conversion of VSMCs remain unclear, recent evidence supports the role of COX2 induction during vascular remodeling. TNFα can induce the expression of COX2 in various cell types^18–22^, and likewise, we found that TNFα stimulates the promoter activity of COX2 and thereby enhances COX2 expression in contractile VSMCs (Fig. 2a–c). Several lines of evidence support the role of COX2 induction by TNFα in contractile VSMC phenotypic changes. First, the silencing or overexpression of COX2 significantly affected smooth muscle marker gene expression and contractile function in VSMCs (Fig. 2d, e, g, h, Supplementary Fig. S3b). Second, the inhibition of COX enzymatic activity suppressed TNFα-induced contractile VSMC phenotypic changes and carotid artery ligation-induced neointima formation (Figs. 2f, 6b). Third, it has been reported that the induction of COX2 by TNFα is required for VSMC proliferation^23^. Therefore, these results suggest that the TNFα-dependent induction of COX2 and COX2 enzymatic activity are required for VSMC phenotypic conversion.

Prostanoids, which consist of prostaglandins and thromboxanes, are produced by COX family members, and our results show that PGD_2_ (a prostaglandin produced by COX2) seems to be a major factor in VSMC phenotypic changes (Fig. 3a, Supplementary Fig. S4a, b). Likewise, it has been reported that lipocalin-type prostaglandin D synthase serum levels are correlated with coronary artery disease severity^38^. Two PGD_2_ receptors, namely, DP_1_ and DP_2_, have been identified thus far^39^, but neither of these two receptors seems to be involved in PGD_2_-mediated VSMC phenotypic changes. For instance, in the present study, neither the inhibition nor activation of DP_1_ and/or DP_2_ affected VSMC phenotypic change (Fig. 3f, g). In line with this, no significant difference in atherosclerosis is found in mice lacking the DP_1_ receptor^40^. In addition, the expression of DP_1_ and DP_2_ was not observed in VSMCs, and the stimulation of VSMCs with PGD_2_ did not alter the levels of cAMP, which is a second messenger of the DP_1_ and DP_2_ receptor signaling pathways (Supplementary Fig. S5). The ERK signaling pathway plays an important role in PGD_2_-induced VSMC phenotypic changes. For example, the stimulation of contractile VSMCs with PGD_2_ strongly activated ERK, and the inhibition of ERK signaling completely blocked PGD_2_-induced VSMC phenotypic changes (Fig. 3c, d), indicating that PGD_2_ induces VSMC phenotypic changes by a DP_1_- or DP_2_-independent mechanism.

There are two mechanistic possibilities for PGD_2_-induced VSMC phenotypic changes: (1) a novel receptor that activates the ERK signaling pathway and (2) the metabolic conversion of PGD_2_. PGD_2_ is metabolized to lipid mediators, such as prostaglandin J_2_ (PGJ_2_), Δ^12^-prostaglandin J_2_ (Δ^12^-PGJ_2_), 15-deoxy-Δ^12,14^-prostaglandin J_2_ (15-d-PGJ_2_), Δ^12^-prostaglandin D_2_ (Δ^12^-PGD_2_), and 13,14-dihydro-15-keto-PGD_2_ (DK-PGD_2_)^29,30^. Indeed, in the present study, PGJ-type PGD_2_ metabolites significantly induced VSMC phenotypic changes (Fig. 4a). PGJs have been reported to be endogenous ligands of PPARδ^32^, and our results also showed that PGJs significantly activated PPRE promoter activity (Fig. 4b), which was also significantly blunted by the inhibition of ERK. It is also noteworthy that PGJs significantly enhanced ERK activity (Supplementary Fig. S6). Furthermore, several of our observations support the role of PPARδ in VSMC phenotypic changes. (1) A selective PPARδ antagonist blunted VSMC phenotypic changes induced by 15-d-PGJ_2_ (Fig. 4c). (2) The ectopic regulation of PPARδ expression significantly affected phenotypic changes in VSMCs (Fig. 4e, f). (3) The overexpression of PPARδ enhanced VSMC proliferation (Fig. 4h). These observations encourage us to suggest that ERK-dependent PPARδ activation is involved in the regulation of VSMC phenotypes.

Since VSMCs also exhibited a decreased expression of PPARγ (Supplementary Fig. S7b), it is possible that PGD_2_-dependent phenotypic changes in VSMCs are mediated by PPARγ. Indeed, the PPARγ isoform showed a similar effect in terms of phenotypic changes in VSMCs (Supplementary Fig. S8). The role of PPARγ in the development of cardiovascular disease is somewhat controversial. For example, the forced expression or disruption of PPARγ has incompatible effects in terms of VSMC proliferation, cardiovascular disease progression, and the mediation of pro- or anti-inflammatory responses^34,41^. Currently, we do not have any direct evidence defining the exact role of PPARγ in cardiovascular disease, but strongly believe that the role of PPARγ is determined by cellular or disease contexts that might be influenced by many factors.

In the present study, we provide evidence that the COX2-dependent generation of PGD_2_ followed by PPARδ activation plays an important role in pathological changes in VSMC phenotypes (Fig. 6d). In this regard, our findings suggest that selective modulation of the biological activity of PGD_2_ might be therapeutically useful in the context of cardiovascular disease.

## Supplementary information

Supplemental Material
