# Supplementary material for: Prostaglandin D2 stimulates phenotypic changes in vascular smooth muscle cells
Source: Exp Mol Med. 2019 Nov 18;51(11):137. doi: 10.1038/s12276-019-0330-3 (PMC6859158; doi:10.1038/s12276-019-0330-3)
Supplement: Supplementary file 1 — Supplemental Material [file 12276_2019_330_MOESM1_ESM.docx]

**Supplemental information**

**Supplemental methods**

*Cell proliferation assay*

VSMCs (1×10^4^ cells/well) were seeded on a six-well plate and grown in the presence or absence of prostanoids for the indicated time points. Cells were fixed with 4% paraformaldehyde, and the nuclei were stained with DAPI. Stained cells were visualized with a fluorescence microscope and random images of four fields were taken at 20× magnification. Results are presented as means ± SEM.

*cAMP measurement*

Contractile VSMCs were plated at 7×10^5^ cells/60-mm culture dishes in complete growth medium, 18-24 h prior to assay. After being serum starved in DMEM basal medium for 24 h, VSMCs were stimulated with 10 μM of PGD_2_ or 10 μM of forskolin for 5 min. After incubation, cells were lysed with 0.1 M HCl for 20min, and intracellular cAMP levels were measured using a cAMP ELISA kit (Cayman Chemical CO., Ann Arbor, MI, USA), according to the manufacturer’s instructions.

*Intracellular Ca^2+^ measurement*

Intracellular calcium concentration was measured using fura-2/AM, a calcium-sensitive fluorescent dye. Briefly, a total of 1X10^6^ cells were incubated with 3 μM fura-2/AM at 37℃ in fresh serum-free DMEM medium with stirring for 50 min. After incubation, cells were washed with serum-free medium twice to remove free dye. Cells (5X10^5^) were aliquoted for each assay into Locke’s solution (154 mM NaCl, 5.6 mML KCl, 1.2 mM MgCl_2_, 5 mM HEPES pH7.3, 10 mM D(+)glucose, 0.2 mM EGTA) for each assay. Fluorescence measurements were performed in a water-jacketed cuvette (37°C) with continuous stirring. During the recording, reagents were added to the cuvette by pausing the recording for 2 sec to open the cuvette-containing chamber. Fluorescent emission at 500 nm was measured at excitation wavelength of 340/380 nm.

**Supplemental results**

*Primary rat aortic smooth muscle cells (RASMCs) were contracted by AngII stimulation*

To assess the feasibility of using established cell lines used to study of VSMC phenotypic change, the expressions of marker genes were verified in established A10 cell lines, primary human carotid artery endothelial cells (HCAECs), and RASMCs. As shown in Fig. S1**a**, S1**b**, both A10 and RASMCs expressed smooth muscle marker genes whereas only HCAECs expressed endothelial marker gene. As shown in Fig. S1**c**, RASMCs contracted in response to AngII stimulation, whereas A10 cells did not.

**
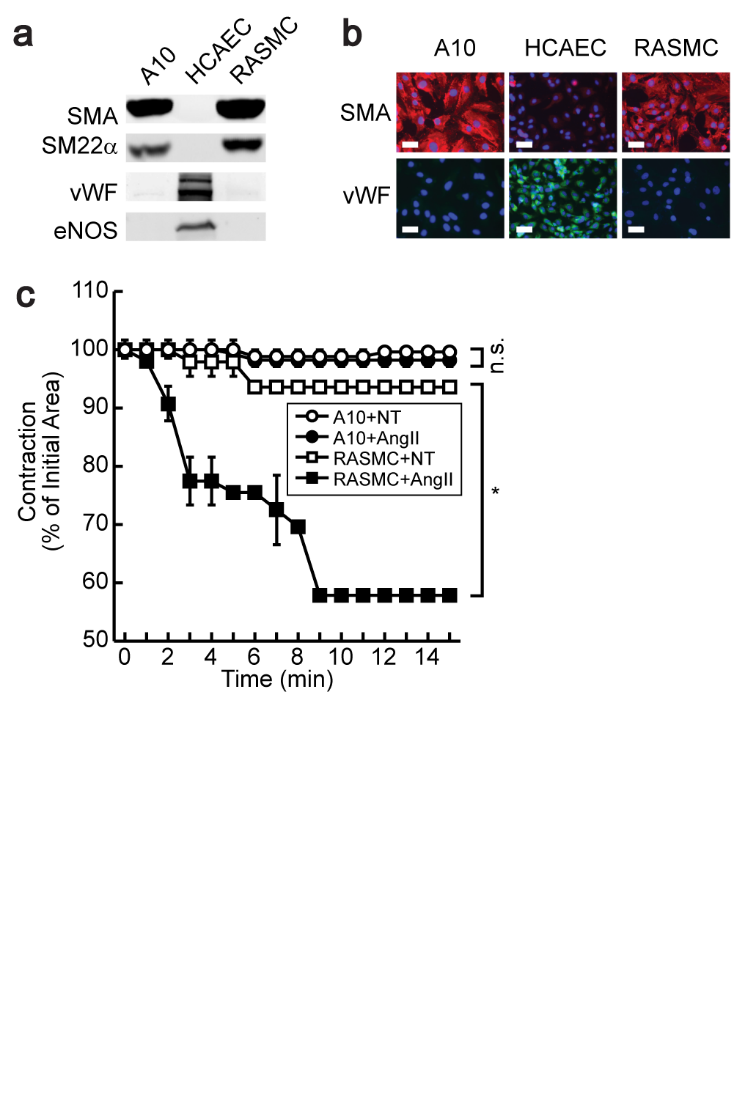
**

**Supplementary Fig. S1** Contraction of RASMCs by AngII stimulation. The expressions of smooth muscle and endothelial cell marker genes were verified by western blot analysis (**a**) and immunocytochemistry (**b**). **Bar,** 200 μm. **c** AngII-induced cell contraction was examined in A10 and RASMCs (P4 stage) (*n*=2). Results are presented as means ± SEM. One-way ANOVA and Tukey’s multiple comparisons test used to produce the P values. Asterisks indicate statistical significance (*P*<0.05).

*TNFα regulated the promoter activities of myocardin, SMA, and SM22α*

As shown in Fig. S2**a**, stimulation of VSMCs with TNFα significantly suppressed the promoter activity of myocardin, and also significantly suppressed the promoter activities of SMA and SM22α (Fig. S2**b**).

**

**

**Supplementary Fig. S2. Suppression of promoter activity of smooth muscle marker gene by TNFα. a** P4 stage VSMCs were transfected with myocardin promoter subcloned in pGL3 vector. Promoter activity of myocardin was confirmed after stimulating cells with TNFα (*n*=3). **b** P4 stage VSMCs were transfected with the promoters of SMA or SM22α. Cells were stimulated with TNFα and promoter activities were measured (*n*=2). Results are presented as means ± SEM. One-way ANOVA and Tukey’s multiple comparisons test used to produce the P values. Asterisks indicate statistical significance (*P*<0.05).

*Silencing of COX2 in VSMCs suppressed the TNFα-induced downregulation of SM22α*

As shown in Fig. S3**a**, COX2 was highly expressed in synthetic type VSMCs, and its expression gradually downregulated during the phenotypic of change of VSMCs from the synthetic to the contractile type. As shown in Fig. S3**b**, TNFα suppressed the promoter activity of SM22α, but this suppression was not observed in COX2 silenced VSMCs.





**Supplementary Fig. S3 Effect of COX2 on the expression of smooth muscle marker gene.** **a** P0 stage VSMCs (synthetic type) were plated on laminin-coated plates and allowed to differentiate into the contractile type. Expressions of COX2 and contractile marker gene were assessed at the indicated times. **b** COX2 was silenced in P4 stage VSMCs and SM22α promoter activity was assessed (*n*=3). Results are presented as means ± SEM. One-way ANOVA and Tukey’s multiple comparisons test used to produce the P values. Asterisks indicate statistical significance (*P*<0.05).

*PGD_2_ induced phenotypic change in VSMCs*

Since COX2 produces various prostanoids, we examined the effects of different prostaglandins on the VSMC phenotypic change. As shown in Fig. S4**a**, both TNFα and PGD_2_ significantly downregulated the expression of SMA and SM22α. However, other prostaglandins or thromboxane had no effect. Furthermore, only PGD_2_ suppressed SM22α promoter activity (Fig. S4**b**). As shown in Fig. S4**c**, PGD_2_ transiently activated ERK whereas p38 MAPK and JNK were marginally activated.





**Supplementary Fig. S4 PGD_2_-dependent VSMC phenotypic change.** **a** P4 stage VSMCs were stimulated with the indicated prostaglandins and the expressions of SMA and SM22α were quantified using Odyssey image software (Li-COR) (*n*=4). **b** P4 stage VSMCs were stimulated with the indicated prostaglandins and SM22α promoter activities were measured (*n*=3). **c** P4 stage VSMCs were stimulated with PGD_2_ and activation of ERK, p38 MAPK, and JNK were assessed at the indicated times. Results are presented as means ± SEM. One-way ANOVA and Tukey’s multiple comparisons test used to produce the P values. Asterisks indicate statistical significance (*P*<0.05).

*cAMP generation and calcium mobilization were not affected by PGD_2_*

Since DP_1_ and DP_2_ receptors (receptor for PGD_2_) are coupled with Gs or Gi protein, we investigated the generation of cAMP by PGD_2_. cAMP levels were unaffected by PGD_2_ under serum-starved conditions (Fig. S5**a**) and normal serum conditions (Fig. S5**b**). To confirm the involvements of DP_1_ and DP_2_ receptors, P4 stage VSMCs were stimulated with DP_1_ (Fig. S5**c**) or DP_2_ (Fig. S5**d**) receptor agonists and cAMP levels were measured. Neither DP_1_ nor DP_2_ receptor agonist affected the levels of cAMP. In addition, both synthetic and contractile VSMCs did not express the DP_1_ and DP_2_ receptors as judged by RT-PCR (Fig. S5**e**). Finally, angiotensin II (AngII) significantly induced calcium mobilization while silencing of PLC-β3 blunts the effect of AngII (Fig. S5**f**-**h**). However, PGD_2_ did not affect calcium mobilization.





**Supplementary Fig. S5 Effect of PGD_2_ on the production of cAMP and calcium mobilization. a** P4 stage VSMCs were serum-starved for 12 h, stimulated with PGD_2_, and cAMP levels were measured. Forskolin (FSK, an adenylyl cyclase activator) was included as a positive control (*n*=3). **b** P4 stage VSMCs was stimulated with PGD_2_ under normal serum conditions, and cAMP levels were measured at the indicated times (*n*=3). **c** P4 stage VSMCs were serum-starved for 12 h and stimulated with a DP_1_-specific agonist (BW245C) (*n*=3). cAMP levels were measured after 30 mins of stimulation. **d** P4 stage VSMCs were stimulated with a DP_2_-specific agonist (DK-PGD_2_) under normal serum conditions. cAMP levels were measured after stimulation for 30 min (*n*=3). **e** mRNAs were isolated from both synthetic and contractile VSMCs, and expression of DP_1_ and DP_2_ receptors was measured by RT-PCR. L6 myocytes were included as positive control. **f** P4 stage VSMCs were pretreated with PGD_2_ and AngII-induced calcium mobilization was measured. PLC-β3 was silenced in P4 stage VSMCs (**g)**, and AngII- or PGD2-induced calcium mobilization was measured (**h**). Results are presented as means ± SEM. One-way ANOVA and Tukey’s multiple comparisons test used to produce the P values. Asterisks indicate statistical significance (*P*<0.05).

*ERK was activated by PGJ series PGD_2_ metabolites*

Since ERK activation is essential for PGD_2_-induced VSMC phenotypic change, we investigated the activation of ERK by PGJs. As shown in Fig. S6, ERK was significantly activated by PGJ_2_, Δ^12^-PGJ_2_, or 15-d-PGJ_2_, but neither Δ^12^-PGD_2_, 15-d-PGD_2_, or DK-PGD_2_ induced ERK activation.





**Supplementary Fig. S6 Activation of ERK by PGJ series PGD_2_ metabolites.** P4 stage VSMCs were stimulated with each PGD_2_ metabolite and ERK activation was verified at the indicated time points.

*VSMCs mainly expressed PPARδ and PPARγ*

Since PGJ series of metabolites significantly induced phenotypic change of contractile VSMCs and are endogenous ligand for PPAR, we next examined the expression of PPAR isoform in VSMCs. As shown in Fig. S7**a**, 7**b**, both synthetic and contractile VSMCs expressed PPARγ and PPARδ but not PPARα. In addition, we used U0126 which is a specific ERK inhibitor to show whether or not it affects PPRE promoter activity. As shown in Fig S7**c**, inhibition of ERK significantly reduced PPARδ-dependent PPRE activity.





**Supplementary Fig. S7 Expression of PPARs in contractile VSMCs. a** Expression of PPARα, PPARδ, and PPARγ was verified in P4 stage VSMCs by RT-PCR. **b** Expression of PPARα, PPARδ, and PPARγ was verified in both synthetic and contractile VSMCs. **c** Contractile VSMCs were infected with retrovirus carrying either vector or PPARδ construct and 15-d-PGJ_2_-dependent promoter activity of PPRE was measured in the presence of ERK inhibitor (U0126, 10 μM) (*n*=3).

*PPARγ facilitated phenotypic change of contractile VSMC.*

As shown in Fig. S8**a**, PPARγ overexpression significantly enhanced contractile VSMC phenotypic change in the presence of rosiglitazone or 15-d-PGJ_2_. Silencing of PPARγ inhibited PGD_2_- or 15-d-PGJ_2_-induced of contractile VSMC phenotypic change (Fig. S8**b**). In addition, the expression of wild type PPARγ facilitated PGD_2_- and 15-d-PGJ_2_-induced contractile VSMC phenotype conversion whereas PPARγ-DM expression had no effect (Fig. S8**c**). Also, overexpression of PPARγ, but not of PPARγ-DM, enhanced contractile VSMC proliferation (Fig. S8**d**), and stimulations of contractile VSMCs with 15-d-PGJ_2_ significantly enhanced proliferation (Fig. S8**e**).





**Supplementary Fig. S8 Effect of PPARγ on the phenotypic change of contractile VSMCs. a** FLAG-tagged PPARγ was expressed in P4 stage VSMCs and the expressions of SMA and SM22α were assessed after stimulation with rosiglitazone or 15-d-PGJ_2_ for 4 days. **b** PPARγ was silenced in P4 stage VSMCs, and the expression of SMA and SM22α were assessed after stimulating cells with PGD_2_ or 15-d-PGJ_2_. **c** Wild type or DNA binding domain mutant (DM) PPARγ were expressed in P4 stage VSMCs, and the expressions of SMA and SM22α were assessed after stimulation with PGD_2_ and 15-d-PGJ_2_. **d** Wild type or DNA binding domain mutant PPARγ was expressed in P4 stage VSMCs, and proliferations were measured. **e** PPARγ was overexpressed in P4 stage VSMCs, and proliferations was measured in the presence or absence of 15-d-PGJ_2_. Results are presented as means ± SEM. One-way ANOVA and Tukey’s multiple comparisons test used to produce the P values. Asterisks indicate statistical significance (*P*<0.05).

**Supplemental Tables**

*Table 1. Antibodies*

| Antibody | Type | Source | Identifier | Dilution |
| --- | --- | --- | --- | --- |
| SMA | Mouse monoclonal | Sigma-aldrich | A2547 | 1:1000 |
| Calponin | Mouse monoclonal | Sigma-aldrich | C2687 | 1:1000 |
| SM22α | Rabbit polyclonal | Abcam | ab14106 | 1:5000 |
| MHC | Rabbit polyclonal | Proteintech | 21404-1-AP | 1:1000 |
| MLCK | Rabbit monoclonal | Abcam | ab76092 | 1:1000 |
| Actin | Mouse monoclonal | Mpbio | SKU 0869100-CF | 1:25000 |
| COX2 | Mouse polyclonal | Cayman | 160106 | 1:200 |
| COX1 | Mouse polyclonal | Santa Cruz | sc-1754 | 1:1000 |
| p-ERK | Rabbit monoclonal | Cell signaling | 4370 | 1:1000 |
| t-ERK | Rabbit monoclonal | Cell signaling | 4695 | 1:1000 |
| MAC2 | Rabbit polyclonal | Santa Cruz | sc-20157 | 1:100 |
| vWF | Rabbit polyclonal | DAKO | A0082 | 1:1000 |
| eNOS | Rabbit polyclonal | Cell signaling | 9572 | 1:1000 |
| pLC-3β | Rabbit polyclonal | Santa Cruz | sc-403 | 1:1000 |

*Table 2. PCR Primers*

| Gene | Gene Assesion | Primer Sense | |
| --- | --- | --- | --- |
| PPARα | NM_013196.1 | F | AGAAGTTGCAGGAGGGGATT |
|  |  | R | CTTCTTGATGACCTGCACGA |
| PPARδ | NM_013141.2 | F | AACATCCCCAACTTCAGCAG |
|  |  | R | TACTGCGCAAGAACTCATGG |
| PPARγ | NM_013124.3 | F | CATTTTTCAAGGGTGCCAGT |
|  |  | R | GAGGCCAGCATGGTGTAGAT |
| DP1 | NM_001135164.1 | F | TACTGGCTATGGCACTAGAGTG |
|  |  | R | GAACACATGGTGAAGAGCACTG |
| DP2 | NM_001012070.1 | F | GGCCGCATCATGTGCTATTACA |
|  |  | R | CCTCAGCTTGTGCAACATGTC |
| SM22α | NM_031549.2 | F | ATCCTATGGCATGAGCCGTG |
|  |  | R | CAGGCTGTTCACCAACTTGC |
| GAPDH | NM_017008.4 | F | AAGGTCGGTGTGAACGGATT |
|  |  | R | CACTTTGTCACAAGAGAAGGCA |

*Table 3. Reagents*

| Reagent |  | Source | Identifier |
| --- | --- | --- | --- |
| TNFα | Tumor necrosis factor-α | KOMA Biotech | K0921049 |
| AngⅡ | Angiotensin II | Sigma-Aldrich | A9525 |
| PGD2 | Prostaglandin D2 | Cayman | 12010 |
| PGE2 | Prostaglandin E2 | Cayman | 14010 |
| PGI2 | Prostaglandin I2 | Cayman | 18220 |
| PGF2α | Prostaglandin F2α | Cayman | 16020 |
| U46619 | Thromboxane receptor agonist | Sigma-Aldrich | D8174 |
| PD98059 | MEK inhibitor | Calbiochem | 513000 |
| SP600125 | JNK inhibitor | Wako | 129-56-6 |
| SB203580 | p38 inhibitor | Calbiochem | 559389 |
| BWA868C | DP receptor antagonist | Cayman | 12060 |
| TM-30089 | CRTH2 receptor antagonist | ChemieTe K | CT-AT002 |
| BW245C | DP1 receptor agonist | Cayman | 12050 |
| DK-PGD2 | DP2 receptor agonist | Cayman | 12610 |
| Δ12-PGD2 | Δ12-Prostaglandin D2 | Cayman | 12650 |
| 15-d-PGD2 | 15deoxyΔ12,14-PGD2 | Cayman | 12700 |
| PGJ2 | Prostaglandin J2 | Cayman | 18500 |
| Δ12-PGJ2 | Δ12-Prostaglandin J2 | Cayman | 18550 |
| 15-d-PGJ2 | 15deoxyΔ12,14-PGJ2 | Cayman | 18570 |
| GW6471 | PPARα antagonist | Sigma-Aldrich | G5045 |
| GSK3787 | PPARδ antagonist | Sigma-Aldrich | G7423 |
| GW9662 | PPARγ antagonist | Sigma-Aldrich | M6191 |
| GW501516 | PPARδ agonist | Enzo | ALX-420-032 |
